# Digital Health Interventions in Physiotherapy: Development of Client and Health Care Provider Survey Instruments

**DOI:** 10.2196/25177

**Published:** 2021-07-28

**Authors:** Mark Merolli, Rana S Hinman, Belinda J Lawford, Dawn Choo, Kathleen Gray

**Affiliations:** 1 Centre for Digital Transformation of Health Faculty of Medicine Dentistry & Health Sciences The University of Melbourne Melbourne, Victoria Australia; 2 Centre for Health, Exercise, and Sports Medicine Department of Physiotherapy, School of Health Sciences Faculty of Medicine Dentistry & Health Sciences, The University of Melbourne Melbourne, Victoria Australia; 3 Department of Audiology and Speech Pathology Faculty of Medicine Dentistry & Health Sciences The University of Melbourne Melbourne, Victoria Australia

**Keywords:** digital health interventions, surveys and questionnaires, World Health Organization, physiotherapy, physical therapy, musculoskeletal, mobile phone

## Abstract

**Background:**

The advancement of digital health has widened the scope of technology use across multiple frontiers of health care services, including personalized therapeutics, mobile health, eHealth record management, and telehealth consultations. The World Health Organization (WHO) responded to this in 2018 by publishing an inaugural broad classification framework of digital health interventions (DHIs) used to address contemporary health system needs.

**Objective:**

This study aims to describe the systematic development of dual survey instruments (clinician and patient) to support data collection, administered in a physiotherapy setting, about perceptions toward DHIs. This is achieved by adapting the WHO framework classification for DHIs for application in real-world research.

**Methods:**

Using a qualitative item review approach, WHO DHI descriptors were adapted and refined systematically to be used in a survey form. This approach was designed to align with the processes of delivering and receiving care in clinical practice, using musculoskeletal physiotherapy as a practical case scenario.

**Results:**

Complementary survey instruments (for health care providers and clients) were developed by adapting descriptor items. These instruments will be used in a larger study exploring the willingness of physiotherapists and patients to use digital technologies in the management of musculoskeletal conditions.

**Conclusions:**

This study builds on the WHO-standardized DHI framework. We developed dual novel survey instruments by adapting and refining the functions of DHIs. These may be deployed to explore the perceived usefulness and application of DHIs for different clinical care functions. Researchers may wish to use these survey instruments to examine digital health use systematically in a variety of clinical fields or technology scenarios in a way that is standardized and generalizable.

## Introduction

### Background

Digital health interventions (DHIs) are increasingly being considered for integration into standard clinical care; in community, primary care; and in hospital settings. This has been accelerated in recent months due to the necessity and urgency to take up DHIs in light of health care pressures and physical distancing necessitated by the COVID-19 pandemic [1-3]. These DHIs may include internet and web-based patient portals, smartphones and apps, electronic health records, decision support tools, wearables, social networks, telehealth consultations, and others [1,4-6]. DHIs appear to offer the possibility of “...effective, cost-effective, safe, and scalable interventions to improve health and healthcare” [4]. Face-to-face or remote models of clinical care may benefit from integrating many varied DHIs to support data collection and analytics of patient examination records, remote monitoring of clinical progress, health status change alerting, delivering teleconsultation, providing web-based education, aiding clinical decision support, or enabling professional-to-professional communication (among others).

### DHIs and Physiotherapy

In the case of physiotherapy (physical therapy) care, routine management of patients presenting with musculoskeletal (MSK) conditions (muscles, ligaments, tendons, joints, nerves, or bones) follows a complex iterative feedback loop of assessment and treatment strategies that generate and test clinical hypotheses and support diagnosis and treatment planning [7,8]. In practice, a physiotherapist collects and analyzes several variables subjectively (qualitative) and objectively (quantitative) about a patient’s presentation to formulate decision-making about treatment strategies and planning [9,10]. In real-world practice, it can be a challenge for physiotherapists and patients to capture and synthesize subjective (qualitative) and objective (quantitative) data consistently and reliably. In this regard, DHIs offer potential in physiotherapy and have garnered increased attention [11]. They offer physiotherapists and patients tremendous potential to streamline and automate data collection, remotely monitor health status, alert and prompt, signal care progress or outcomes, and deliver consultations [4,12,13].

Digitally supported care has great potential to positively impact patient management, health behaviors, and treatment satisfaction, which contribute to better health outcomes and improve health system performance [14]. DHIs that are scalable can also overcome barriers to access or cost and may be individualized to meet patient and clinician needs and expectations [4,6]. Nonetheless, evidence about DHI acceptance, uptake, and efficacy remains limited [15], partly because DHIs are complex and multifaceted and because of the lack of a standardized DHI classification system that is easily grasped by both patients and health care providers (HCPs) [4]. Furthermore, although research investigating the acceptance of DHIs exists, assessment of engagement with digital health requires more diverse and comprehensive measures of evaluation than those that currently exist [4,6,15].

### Digital Health Classifications

In 2018, the World Health Organization (WHO) released the *Classification of Digital Health Interventions v1.0* [5]. This framework provides a way to describe and classify how digital health technologies are being leveraged by stakeholders across health systems. Inherently, the framework offers a mechanism for stakeholders (such as researchers, HCPs, clients [patients], vendors, and policy makers) to describe the numerous functions of digital health. The WHO’s DHIs framework spans a repertoire of mobile health, eHealth, and emerging technological capabilities, which may be used to address health system challenges. Each intervention is paired with associated synonyms and illustrative examples of digital health use. The DHIs are also grouped and mapped to functions relevant to each of the 4 targeted primary user groups (clients, HCPs, health system or resource managers, and data service users) [5]. This framework, which describes DHIs in a common uniform language, is a major advance toward supporting the standardized, accurate, and reliable reporting of DHIs in scientific research, in clinical record-keeping and communications, and in service improvement projects [5,16].

There are, however, potential barriers to the broader application of the DHI framework. These include its predominant focus on public health, as attested to by the WHO [5]. It may not adequately take into account health care delivered in private sector settings. The language used to describe various functions of digital health is arguably technical, and the terminology appears framed more for the health informatics community rather than a clinical community that may possess less advanced digital health competency. Tensions around language and vocabulary used in health informatics continue to be discussed and have been the focus of debate in the scholarly community for some time [17,18]. Thus, the framework in its current form may be most accessible to those with specific backgrounds in digital health and informatics, limiting its potential broader application among HCPs and clients directly involved in clinical care. For example, the language used to describe digital health use by HCPs around structured versus unstructured records may not be easily understood. The following examples come from the WHO classifications of DHIs for HCPs:

2.2.2 Manage a client’s structured clinical records

2.2.3 Manage a client’s unstructured clinical records

Using a less formal, discipline-specific language might help individuals to understand or quantify the unique meaning of discrete functions of digital health. Furthermore, augmenting this work with a view toward supporting data collection to gather individual perceptions about DHIs may allow increased real-world adoption of the WHO framework in clinical research. In addition, this provides a way to not only view DHIs through the lens of a taxonomy but also to quantify engagement with digital health.

Hence, the objectives of this paper are to develop dual (clinician and patient) survey instruments that support data collection about perceptions toward willingness to engage with DHIs, administered in a physiotherapy setting as a case study. This was achieved by adapting the WHO’s framework classification for DHIs. This work may support the increased real-world adoption of the WHO framework in further clinical research settings.

## Methods

### Clinical Case Study in Physiotherapy: Developing New Survey Instruments

Our case study setting applies the translated DHI functions to a pair of surveys developed for use within the context of physiotherapy (physical therapy) and MSK health conditions. MSK conditions are health problems related to muscles, ligaments, tendons, joints, nerves, or bones. This realm was identified as a suitable case study because it is the professional background of 3 of the chief investigators (MM, RSH, and BJL); their reputations and networks increased the likelihood of being able to recruit participants in phase 4. The survey instruments were designed to measure *willingness* of clients and physiotherapists to engage with DHIs in real-world scenarios.

### Qualitative Item Review of WHO Framework Items to Inform Survey Instruments in a Physiotherapy Setting

#### Overview

The *Results* section provides a rationale for the use of qualitative item review (QIR) [19] to adapt the WHO DHI items for inclusion in a pair of surveys. The authors followed widely accepted systematic steps in developing the survey tools, as detailed in previous well-regarded and highly cited papers [20,21]. Furthermore, we highlight select target end user feedback to support the refinement of the final items to be included in the survey instruments. Ethical approval was granted by the Human Research Ethics Committee of the University of Melbourne (study ID 2056217.1).

#### Rationale

The method used to adapt and refine descriptor items in the WHO classification of DHIs framework leverages QIR. We used an MSK physiotherapy case study example to outline the process. This is based on previous work by the authors published in this journal [22], the authors’ clinical profiles, and their informatics research backgrounds. QIR has previously been used by the National Institutes of Health in their Patient-Reported Outcome Measurement Information System to develop several patient-reported outcome measure item banks to create standardized patient-reported outcome measure surveys for research across various health areas [19]. The QIR method is appropriate as it allows for the identification and scrutiny of items, which are further optimized by expert review and through the elicitation of target end user feedback [22].

#### Procedures

**Figure 1:**
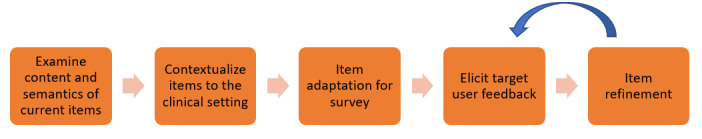
A systematic approach to qualitative item review and adaptation of items in the World Health Organization Classification of Digital Health Interventions v1.0.

The QIR method was applied to the original set of WHO DHI Clients 1.0 and Health care Providers 2.0 items. An iterative approach over five phases was conducted, led by 2 of the primary study authors (Figure 1). This was reviewed by the rest of the research team to verify the accuracy and alignment against the original WHO framework, until consensus was reached on the final item for inclusion in our survey. The phases were as follows: (1) examine the connotations of the original top-level DHI function and consider the semantics of each item’s wording, (2) contextualize items to the clinical setting, (3) adapt item wording into a survey instrument format, (4) elicit target end user feedback, and (5) final refinement of survey item.

A tracking system was developed to record any adaptations made to items using a Microsoft Excel spreadsheet defined by a data coding version (iteration) number. All coauthors have professional backgrounds in either the allied health clinical sciences and clinical informatics (eg, physiotherapy, speech pathology, health science, and health informatics). Furthermore, all coauthors have extensive prior research experience in the development of survey instruments for HCPs and clients.

#### Phases 1 and 2

During the first two phases, the content and semantics of each existing WHO DHI descriptor were assessed, contextualized, and adapted where deemed appropriate by 3 authors separately and then together (including the primary author), to reflect the specific nature of patient care in the clinical context (eg, MSK physiotherapy). Original descriptor domains were rephrased in lay terms in an attempt to describe the various functions of DHIs to support care (ie, HCPs delivering care vs clients or patients managing conditions). There were 10 HCP functions, with 32 items to examine, and 7 client functions, with 16 items to examine. The rest of the research team discussed the appropriateness and interpretation of the revised DHI functions for fit until an intercoder agreement for each descriptor was established.

#### Phase 3

During phase 3, 2 authors (including the primary author) transformed items into survey instrument question stems that framed items to assess *willingness* to use the DHIs for various purposes, as categorized in the WHO framework. The result is the direct product of adapting the WHO framework items into a question form, not from some preexisting questionnaire.

#### Phases 4 and 5

In phase 4, a convenience sample of target end users (9 physiotherapists [HCPs]) working in MSK clinical practice and 11 physiotherapy service users (clients) were invited to review the adapted survey items and provide feedback on their clarity, comprehension, and validity for final inclusion in the survey instruments. Participants’ demographic characteristics are provided in Multimedia Appendix 1. These participants were invited to provide written free-text comments about their ability to interpret the survey items and language used. Three authors (including the primary author) collated, analyzed, and discussed the feedback. This process allowed for a consensus about any items requiring further revision. Items were then further revised in a final iteration during phase 5, which were revisited with the target users to confirm the fit for survey instrument readiness.

## Results

### QIR of WHO Framework Items to Inform Survey Instruments in a Physiotherapy Setting

#### Phases 1 and 2

Tables 1 and 2 show the original and adapted versions of the WHO framework items for surveying clients and HCPs, respectively. The full refinement process and all item revisions are presented in Multimedia Appendices 2 and 3. All the 16 items of the WHO *Clients* section of the framework were adapted from their original wording. On average, each item was revised 3.3 times before inclusion in the survey.

Of the 32 items in the WHO *Health care Providers* section of the framework, only 1 item (*2.10 Laboratory and Diagnostic Imaging* category—*Capture diagnostic results from digital devices*) was not adapted from its original. On average, the remaining items were revised 3.2 times.

**Table 1:** Final adaptation of the World Health Organization classification items-clients.

Original		Adapted	
Digital health intervention category	Item	Digital health intervention category	Item after QIR^a^
1.1 Targeted client communications	Transmit health event alerts to specific population groups	1.1 Targeted communications	Send me urgent health alerts that people living with my condition need to know (eg, medication product recalls, etc)
N/A^b^	Transmit targeted health information to clients on health status or demographics	N/A	Send me health information of interest to people living with condition (eg, about new treatments, research, etc)
N/A	Transmit targeted alerts and reminders to clients	N/A	Send personalized alerts and reminders relevant specifically to me (eg, about services I’ve booked or have coming up)
N/A	Transmit diagnostic results, or availability of results to clients	N/A	Sends me health test results, or tells me results are available
1.2 Untargeted client communication	Transmit untargeted health information to an undefined population	1.2 General communications	Send me general news or information about good health or healthy living
N/A	Transmit untargeted health event alerts to undefined group	N/A	Send me general health alerts (eg, about environmental factors impacting my ability to exercise-weather, air quality, etc)
1.3 Client to client communication	Peer group for clients	1.3 Person to person communications	Communicate online with other peer groups of people living with my condition
1.4 Personal health tracking	Access by client to own medical records	N/A	Access my own medical records
N/A	Self-monitoring of health or diagnostic data by clients	N/A	Self-monitor my condition or diagnosis-related information
N/A	Active data capture/documentation by clients	N/A	Actively collect information about my condition or injury status and record it
1.5 Citizen-based reporting	Reporting of health system feedback by clients	N/A	Allow me to collect and provide feedback about the health system
N/A	Reporting of public health events by clients	N/A	Allow me to report urgent public health events/issues that people living with my condition need to know
1.6 On-demand information services to clients	Client look-up of health information	1.6 Information when I need it	To look up health information
1.7 Client financial transactions	Transmit or manage out of pocket payments by clients	1.7 Financial transactions	Send or manage any “out of pocket” payments I may need to pay
N/A	Transmit or manage vouchers to clients for health services	N/A	Send or manage vouchers/coupons I might have for health services (eg, travel vouchers)
N/A	Transmit or manage incentives to clients for health services	N/A	Send or manage rewards or incentives I have to use health services

^a^QIR: qualitative item review.

^b^N/A: not applicable.

**Table 2:** Revision of the World Health Organization classification items-health care providers.

Original		Adapted	
Digital health intervention category	Item	Digital health intervention category	Item after QIR^a^
2.1 Client Identification & Registration	Verify client unique identity	2.1 Patient identification & registration	Verify a patient’s personal details (eg, new patient registration)
N/A^b^	Enrol client for a health service or care planning activity	N/A	Make a clinical appointment
2.2 Client Health Records	Longitudinal tracking of client’s health status & services	2.2 Patient Health Records	Track a patient’s condition and/or clinical service use over time
N/A	Manage the client’s unstructured clinical records	N/A	Enter a patient’s free-text clinical progress notes
N/A	Manage the client’s structured clinical record	N/A	Record or code a patient’s condition using standardized coding, checkboxes, and dropdown menus
N/A	Routine health indicator data collection & management	N/A	Record and/or flag indicators of change in a patient’s condition
2.3 Healthcare Provider Decision Support	Provide prompts and alerts according to a specific protocol	2.3 Clinician Decision-Support	Prompt my thinking using software that supports clinical decision-making
N/A	Provide a checklist according to a specific protocol	N/A	Provide me a digital checklist of clinical procedures to follow
N/A	Screen clients by risk or, other health status	N/A	Screen my patients
2.4 Telemedicine	Consultations between remote client and healthcare provider	N/A	Conduct remote consultations
N/A	Remote monitoring of client health, or diagnostic data by healthcare provider	N/A	Remotely monitor or track a patient’s condition
N/A	Transmission of health-related data to healthcare provider	N/A	Send me data about my patient’s condition
N/A	Consultations for case-management between healthcare providers	N/A	Conduct case consultations with other clinicians
2.5 Healthcare Provider Communication	Communication from healthcare providers to supervisor	2.5 Clinician communications	Communicate with a manager or supervisor
N/A	Communication and performance feedback to healthcare providers	N/A	Provide me with feedback about my clinical performance
N/A	Transmit routine news and workflow notifications to healthcare provider	N/A	Send me routine updates and workflow notifications
N/A	Transmit non-routine health event alerts to healthcare providers	N/A	Send non-routine or unexpected health event alerts about a patient
N/A	Peer-group for healthcare providers	N/A	Utilise online peer communication groups for clinicians
2.6 Referral Coordination	Coordinate emergency response and transport	N/A	Coordinate emergency responses and/or transport for a patient
N/A	Manage referrals between points of service with health sector	N/A	Manage health services referrals or reports (eg, to other clinicians)
N/A	Manage referrals between health and other sectors	N/A	Manage referrals or reports to external bodies eg, government services
2.7 Health Worker Activity Planning & Scheduling	Identify clients in need of services	2.7 Clinician workflow coordination	Identify patients in need of a health service
N/A	Schedule healthcare provider’s activities	N/A	Schedule my clinical activities
2.8 Healthcare Provider Training	Provide training content to healthcare providers	2.8 Clinician training	Provide me with training or educational content
N/A	Assess capacity of healthcare providers	N/A	Assess my clinical capacity, or performance
2.9 Prescription & Medication Management	Transmit or track prescription orders	N/A	Track prescription orders
N/A	Track clients’ medication consumption	N/A	Track patient’s medication consumption
2.10 Laboratory & Diagnostic Imaging Management	Report adverse drug events	2.10 Pathology & Imaging Management	Report adverse medication events
N/A	Transmit diagnostic result to healthcare providers	N/A	Send me diagnostic imaging results (eg, scans)
N/A	Transmit and track diagnostic orders	N/A	Track diagnostic imaging orders
N/A	Capture diagnostic results from digital devices	N/A	Capture diagnostic results from digital devices
N/A	Track biological specimens	N/A	Track pathology eg, blood tests

^a^QIR: qualitative item review.

^b^N/A: not applicable.

#### Phase 3

As previously outlined, 2 authors transformed individual items based on their suitability to interpret them in a survey to assess the willingness to use DHIs. Survey questions were framed from the question stem, “How willing are you to use digital technology to...” Response options were provided using a 5-point Likert scale ranging from *not at all willing* to *very much willing*.

#### Phases 4 and 5

Target end user feedback revealed that 14 items—that is, 25% (8/32) of *health care provider* items and 38% (6/16) of *client* items—were flagged by participants as most difficult to interpret. Participants noted challenges in interpreting the meaning of the item in question and issues with the chosen language. In the client items, these were mostly related to domain 1.1: *Targeted communications* and domain 1.2: *General communications* (Table 1). In the HCP items, there were no particular domains (other than domain 2.2: *Client health records* and domain 2.3: *Health care provider decision support*) where difficulties were more commonly reported (Table 2). On the basis of the summative feedback, clients preferred more person-centric language to be used (eg, changing *1.4: Access by client to own medical records* to *Access MY own medical records*), whereas HCPs preferred language to be more clinically focused. These end users preferred *health care providers* to be referred to as *clinicians* and *clients* to be described as *patients*. Furthermore, based on feedback, examples were added to select items (Tables 1 and 2) to provide greater context and to assist with interpretation.

**Figure 2:**
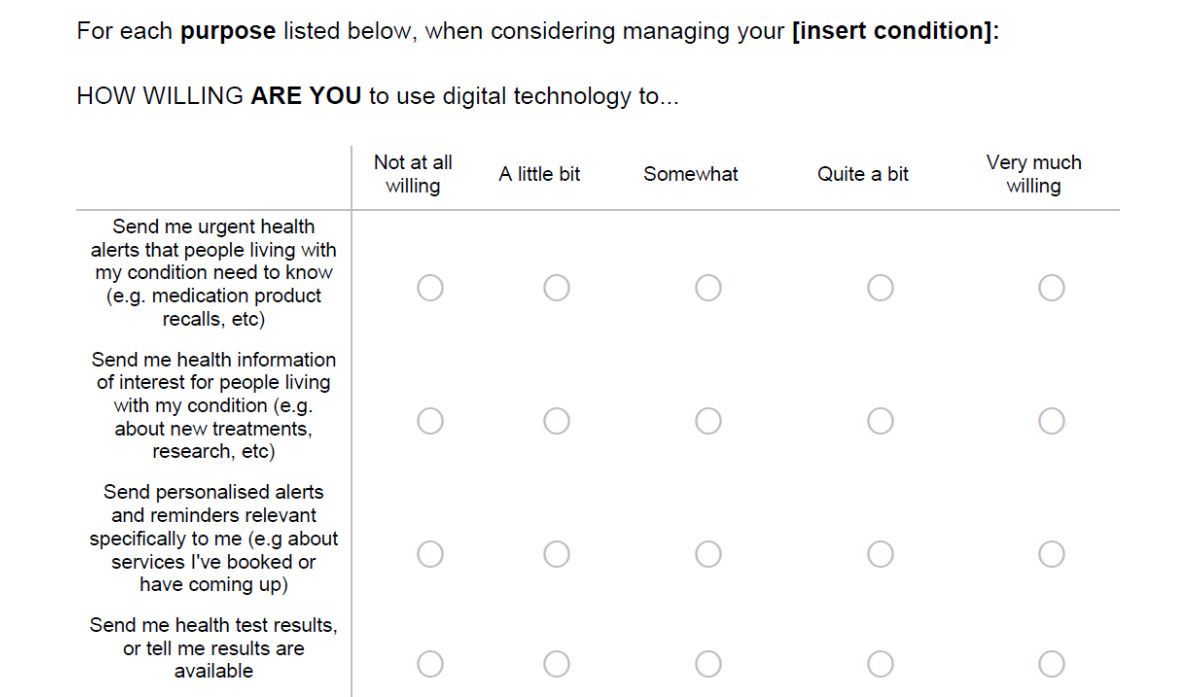
Excerpt from the Digital Health Intervention Willingness-Client survey.

**Figure 3:**
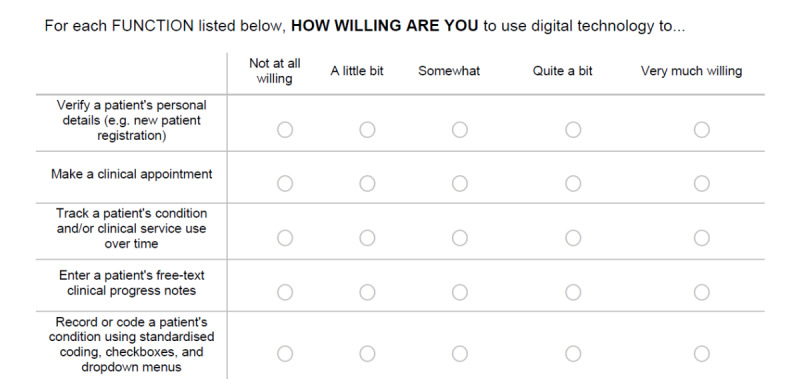
Excerpt from the Digital Health Intervention Willingness-Health care Provider survey.

Samples of both the refined client and HCP instruments are shown in Figures 2 and 3, respectively. The dual survey instruments were named (1) Digital Health Intervention Willingness-Client (DHIW-C; Figure 2) and (2) Digital Health Intervention Willingness-Health care Provider (DHIW-HCP; Figure 3) in the spirit of the original WHO classifications. Both instruments are available in their entirety in Multimedia Appendices 4 and 5. Furthermore, both instruments (Figures 2 and 3) contain person-centered language, such as “How willing are ‘you’...” and “...send ‘me’ urgent...” In both instances, this language refers to the respondent and is deliberately framed in this way to avoid diverging too far from the original WHO framework items. This phraseology was not identified as problematic during piloting, with participants readily identifying “you” and “me” as the same person.

## Discussion

### Principal Findings

This study describes an iterative, phased process undertaken to build on work that classifies DHIs, by adapting items from the WHO classification of DHIs v1.0 for use in dual survey instruments [5]. To our knowledge, this has not been done previously. Findings from this study highlight the complexities of knowledge representation within digital health taxonomies and in the digital health environment. Riaño et al [23] suggested that knowledge representation in health is a complex area, particularly as the domain evolves in the face of advances in technology, such as artificial intelligence. The WHO’s DHI framework is a significant contribution to digital health and informatics research and practice reporting, via its conceptualization of the various functions of DHIs for reporting purposes [5]. We have outlined our approach to build on this and adapt DHI classification items into survey instruments that enable collection of data about willingness to use various functions of digital health. Our aim was to make the various functions of DHIs more clinically applicable in real-world settings for individuals with less advanced knowledge of the digital health ecosystem. This includes both HCPs and consumers as well as clinical researchers wanting to build insights into the use of DHIs from a more evidenced-based perspective.

### Scalable Assessment of Digital Health

Research in the realm of digital health uptake and acceptance suggests that stakeholders in digital health (ie, HCPs, clients, and researchers) recognize the importance of digital health but require more consistent and reliable methods of reporting and evaluating engagement with digital health initiatives [6,15,16]. Various digital health survey instruments exist to examine users’ perceptions of digital health. However, many tend to focus more explicitly on quality and the disparate functions of DHIs. For example, the DISCERN instrument assesses the quality of web-based health information, and the health on the net code is a checklist used to signal quality-controlled health website content (such as web-based patient education materials) [24,25]. Comparably, the Mobile App Rating Scale is specific to surveying and rating mobile apps [26]. In a similar vein, our newly developed surveys (DHIW-C and DHIW-HCP) fill a unique gap that allows examination of user engagement with the various *functions* of digital health, rather than focusing on a single specific category of technology or technological tools.

### Clinical Implications

This study has several clinical implications. First, the corresponding survey instruments may help bridge the current evidence gaps in the reporting of DHIs. Murray et al [4], in their seminal piece on DHI evaluation, stated that DHIs have tremendous potential in terms of scalability for improving health. However, the authors contended that DHIs are complicated to examine and that a stronger, more reliable evidence base is needed to report such interventions [4]. A recent systematic review assessing the efficacy of DHIs to improve health outcomes in the workplace found a modest net effect of DHIs. However, the authors noted the continued highly heterogeneous nature of digital health research as well as difficulties in examining digital health use because of the lack of standardized measures for reporting on key active components of DHIs [27]. The authors concluded that focusing on engagement with DHIs and using standardized measures for describing DHIs will benefit future research and possibly provide greater opportunities for meta-analyses of DHI outcomes [27]. This is further supported by Zanaboni et al [28], who advise that to build a reliable evidence base about digital health use and health outcomes, greater focus needs to be placed on clinical research in the form of high-quality randomized controlled trials. Blandford et al [29] propose that the evaluation of DHIs in research requires flexibility and adaptation of traditional health research methods. For instance, randomized controlled trials investigating DHIs should look to move away from the current approach of assessing the technology *features* (which may be outdated by the time the research is complete) and instead focus on the digital health *principles* latent in the intervention [29]. Our novel measures support this notion, by providing a way to measure perception toward the *functions* of DHIs that lie at the heart of any digital intervention. Furthermore, as seen in the structure of the DHIW-C and DHIW-HCP instruments (Figures 2 and 3), the measures are readily transferrable to a variety of clinical health contexts or specialty areas and conditions, which also allows for comparing and contrasting DHIs.

### Limitations

This study had some limitations. First, the process undertaken using a single discipline case study pertains to a narrow subset of clinical informatics research, limiting its generalizability (ie, a study of DHIs in an MSK physiotherapy context). Other health care professionals and clients with different health problems may have had alternative views on the wording of the items in our surveys. Second, we piloted survey items in phase 4, once items had been adapted, refined, and placed in a question format. It is possible that if target users reviewed items during the earlier phases, opinions may have differed. Involving the target users in phase 4 may be a limitation, rather than involvement in phase 1. However, we felt it prudent to first examine the items and place them in a format that would be meaningful to end users. As their primary input was to comprehend the questions in the surveys, we felt this was the most methodologically sound time to involve them. Furthermore, this study did not aim to assess the psychometric properties of the developed survey instruments. Future research is warranted to assess the instruments’ psychometric properties. Finally, our instruments are presently available only in English, potentially limiting wider global use. However, pending future adaptation and psychometric testing, future research to translate the DHIW-C and DHIW-HCP may be a useful undertaking.

### Conclusions

This study has detailed the systematic development of dual (patient and clinician) survey instruments (DHIW-C and DHIW-HCP) to support data collection about perceptions toward DHIs administered in a physiotherapy setting. This was achieved by adapting the WHO framework classification for DHIs for application in real-world research. It also highlights the applicability of the WHO’s standardized DHI framework in digital health research. With further research, using our surveys as the foundation, future surveys may be developed across a range of health care and technology contexts to examine user perceptions toward willingness to engage with DHIs. As DHIs continue to evolve and their omnipresence grows in research and practice, standardized surveys may be helpful by allowing users to capture information about a broad spectrum of DHI functions that are increasingly prevalent in clinical practice.

## Appendix

Multimedia Appendix 1 Pilot participants’ characteristics.

Multimedia Appendix 2 Data recoding the World Health Organization classifications for survey-client.

Multimedia Appendix 3 Data recoding the World Health Organization classifications for survey-health care professional.

Multimedia Appendix 4 Digital Health Intervention Willingness-Client.

Multimedia Appendix 5 Digital Health Intervention Willingness-Health care Provider.

## Abbreviations

DHI: digital health intervention
DHIW-C: Digital Health Intervention Willingness-Client
DHIW-HCP: Digital Health Intervention Willingness-Health care Provider
HCP: health care provider
MSK: musculoskeletal
QIR: qualitative item review
WHO: World Health Organization

## References

[1] Greenhalgh T, Wherton J, Shaw S, Morrison C (2020). Video consultations for covid-19. Br Med J.

[2] Dantas LO, Barreto RP, Ferreira CH (2020). Digital physical therapy in the COVID-19 pandemic. Braz J Phys Ther.

[3] Tanne JH, Hayasaki E, Zastrow M, Pulla P, Smith P, Rada AG (2020). Covid-19: how doctors and healthcare systems are tackling coronavirus worldwide. Br Med J.

[4] Murray E, Hekler EB, Andersson G, Collins LM, Doherty A, Hollis C, Rivera DE, West R, Wyatt JC (2016). Evaluating digital health interventions: key questions and approaches. Am J Prev Med.

[5] (2018). Classification of Digital Health Interventions. World Health Organisation.

[6] (2019). Summary of Findings and GRADE Tables. World Health Organisation. World Health Organisation.

[7] Edwards I, Jones M, Carr J, Braunack-Mayer A, Jensen G (2004). Clinical reasoning strategies in physical therapy. Phys Ther.

[8] Higgs J, Jones M, Loftus SC (2018). Clinical Reasoning in the Health Professions E-Book.

[9] Atkinson H, Nixon-Cave K (2011). A tool for clinical reasoning and reflection using the international classification of functioning, disability and health (ICF) framework and patient management model. Phys Ther.

[10] Cooper P (2014). Data, information, knowledge and wisdom. Anaesth Intensive Care Med.

[11] Kloek CJ, Janssen J, Veenhof C (2020). Development of a checklist to assist physiotherapists in determination of patients' suitability for a blended treatment. Telemed J E Health.

[12] Slater H, Dear BF, Merolli MA, Li LC, Briggs AM (2016). Use of eHealth technologies to enable the implementation of musculoskeletal Models of Care: Evidence and practice. Best Pract Res Clin Rheumatol.

[13] Chehade MJ, Yadav L, Kopansky-Giles D, Merolli M, Palmer E, Jayatilaka A, Slater H (2020). Innovations to improve access to musculoskeletal care. Best Pract Res Clin Rheumatol.

[14] (2017). Australia’s National Digital Health Strategy- Safe, Seamless and Securevolving Health and Care to Meet the Needs of Modern Australia. Australian Digital Health Agency.

[15] Greenhalgh T, Wherton J, Papoutsi C, Lynch J, Hughes G, A'Court C, Hinder S, Fahy N, Procter R, Shaw S (2017). Beyond adoption: a new framework for theorizing and evaluating nonadoption, abandonment, and challenges to the scale-up, spread, and sustainability of health and care technologies. J Med Internet Res.

[16] Klein G (2018). Standardization of health informatics - results and challenges. Yearb Med Inform.

[17] Ozbolt J (1999). The Vocabulary of Health Informatics For the New Millennium. Biomedical and Health Informatics Conference.

[18] Schulz S, Balkanyi L, Cornet R, Bodenreider O (2013). From concept representations to ontologies: a paradigm shift in health informatics?. Healthc Inform Res.

[19] DeWalt DA, Rothrock N, Yount S, Stone AA, PROMIS Cooperative Group (2007). Evaluation of item candidates: the PROMIS qualitative item review. Med Care.

[20] Boynton PM, Greenhalgh T (2004). Selecting, designing, and developing your questionnaire. Br Med J.

[21] Artino AR, La Rochelle JS, Dezee KJ, Gehlbach H (2014). Developing questionnaires for educational research: AMEE Guide No. 87. Medical Teacher.

[22] Dimaguila GL, Gray K, Merolli M (2020). Patient-reported outcome measures of utilizing person-generated health data in the case of simulated stroke rehabilitation: development method. JMIR Res Protoc.

[23] Riaño D, Peleg M, Ten Teije A (2019). Ten years of knowledge representation for health care (2009-2018): topics, trends, and challenges. Artif Intell Med.

[24] Kaicker J, Borg Debono V, Dang W, Buckley N, Thabane L (2010). Assessment of the quality and variability of health information on chronic pain websites using the DISCERN instrument. BMC Med.

[25] Boyer C, Gaudinat A, Hanbury A (2017). Accessing Reliable Health Information on the Web: a Review of the HON Approach. 16th World Congress on Medical and Health Informatics.

[26] Stoyanov SR, Hides L, Kavanagh DJ, Zelenko O, Tjondronegoro D, Mani M (2015). Mobile app rating scale: a new tool for assessing the quality of health mobile apps. JMIR Mhealth Uhealth.

[27] Howarth A, Quesada J, Silva J, Judycki S, Mills PR (2018). The impact of digital health interventions on health-related outcomes in the workplace: a systematic review. Digit Health.

[28] Zanaboni P, Ngangue P, Mbemba GI, Schopf TR, Bergmo TS, Gagnon M (2018). Methods to evaluate the effects of internet-based digital health interventions for citizens: systematic review of reviews. J Med Internet Res.

[29] Blandford A, Gibbs J, Newhouse N, Perski O, Singh A, Murray E (2018). Seven lessons for interdisciplinary research on interactive digital health interventions. Digit Health.

