# Supplementary material for: Digital Health Interventions in Physiotherapy: Development of Client and Health Care Provider Survey Instruments
Source: JMIR Res Protoc. 2021 Jul 28;10(7):e25177. doi: 10.2196/25177 (PMC8367153; doi:10.2196/25177)
Supplement: Multimedia Appendix 1 [file resprot_v10i7e25177_app1.pdf]

Appendix 1. Pilot participant characteristics

Pilot participant	Age range (years)	Sex	Primary condition (treated or, received) ***Physios treating variety = multiple**
Physio1	30-39	F	Pelvis/hip
Physio2	50-59	M	Low back/buttock
Physio3	60-69	F	Shoulder
Physio4	20-29	M	Multiple
Physio5	30-39	F	Multiple
Physio6	30-39	M	Multiple
Physio7	30-39	M	Multiple
Physio8	40-49	M	Ankle/foot
Physio9	40-49	F	Multiple
Patient1	30-39	F	Low back/buttock
Patient2	60-69	F	Pelvis/hip
Patient3	60-69	F	Knee
Patient4	30-39	M	Low back/buttock
Patient5	20-29	F	Ankle/foot
Patient6	70+	M	Multiple
Patient7	20-29	M	Knee
Patient8	60-69	F	Multiple
Patient9	30-39	F	Multiple
Patient10	70+	F	Ankle/foot
Patient11	40-49	F	Shoulder
