# Supplementary material for: Digital Health Interventions in Physiotherapy: Development of Client and Health Care Provider Survey Instruments
Source: JMIR Res Protoc. 2021 Jul 28;10(7):e25177. doi: 10.2196/25177 (PMC8367153; doi:10.2196/25177)
Supplement: Multimedia Appendix 2 [file resprot_v10i7e25177_app2.pdf]

Appendix 2. Functions of DHI-Clients RECODED for COMPREHENSION

ORIGINAL		RE-CODED	V1	V2	V3	V4	V5
**1.1 Targeted client communication**	Transmit health event alerts to specific population groups	**1.1 Targeted communications**	Send health alerts to specific populations	Sends health alerts to specific groups of people	Send urgent health alerts that people living with my condition need to know	Send me urgent health alerts that people living with my condition need to know (e.g. medication product recalls, etc)	
	Transmit targeted health information to clients on health status or demographics		Send targeted health information to me	Send targeted health information to me	Send health information of interest to people living with condition	Send me health information of interest to people living with condition (e.g about new treatments, research, etc)	
	Transmit targeted alerts and reminders to clients		Send targeted alerts and reminders to me	Send targeted health alerts and reminders to me	Send targeted alerts and reminders relevant specifically to me	Send personalised alerts and reminders relevant specifically to me (e.g about services I've booked or have coming up)	
	Transmit diagnostic results, or availability of results to clients		Send diagnostic test results, or availability of results	Sends me health test results, or tells me results are available			
**1.2 Untargeted client communication**	Transmit untargeted health information to an undefined population	**1.2 General communications**	Send general health information	Send me general health information	Send me general news or information about good health	Send me general news or information about good health or healthy living	
	Transmit untargeted health event alerts to undefined group		Send general health alerts	Send me general health alerts	Send me general health alerts (e.g. about environmental factors impacting my ability to exercise- weather, air quality, etc)		
**1.3 Client to client communication**	Peer group for clients	**1.3 Person to person communications**	Online peer to peer communication and support groups	Exchange online peer communication with groups living with my condition	Communicate online with other peer groups of people living with my condition		
**1.4 Personal health tracking**	Access by client to own medical records	**1.4 Personal health tracking**	Access to my own medical records	Access my own medical records			
	Self-monitoring of health or diagnostic data by clients		Self-monitoring of my health or diagnosis-related information	Self monitor my health or diagnosis-related information	Self monitor my condition or diagnosis-related information		
	Active data capture /documentation by clients		Ability to actively collect health metrics or information and document it	To actively collect information about my condition's status and document it	Actively collect information about my condition's status and document it	Actively collect information about my condition or injury status and document it	Actively collect information about my condition or injury status and record it
**1.5 Citizen-based reporting**	Reporting of health system feedback by clients	**1.5 Citizen-based reporting**	Reporting feedback about the health system by people like me	Collect and provide feedback about the health system by people like me	Collect and provide feedback about the health system by people like me	Allow me to collect and provide feedback about the health system	
	Reporting of public health events by clients		Reporting of public health events by people like me	Report public health events by people like me	Report urgent public health events by that people living with my condition need to know	Allow me to report urgent public health events/issues that people living with my condition need to know	
**1.6 On-demand information services to clients**	Client look-up of health information	**1.6 Information when I need it**	I can look up health information	To look up health information			
**1.7 Client financial transactions**	Transmit or manage out of pocket payments by clients	**1.7 Financial transactions**	Send or manage out of pocket payments	Send or manage any 'out of pocket' payments	Send or manage any 'out of pocket' payments I may need to pay		
	Transmit or manage vouchers to clients for health services		Send or manage vouchers for health services	Send or manage vouchers for health services (e.g travel vouchers)	Send or manage vouchers/coupons I might have for health services (e.g travel vouchers, etc)		
	Transmit or manage incentives to clients for health services		Send or manage rewards or incentives for health services	Send or manage rewards or incentives for using health services	Send or manage rewards or incentives I have to use health services		
