# Supplementary material for: Digital Health Interventions in Physiotherapy: Development of Client and Health Care Provider Survey Instruments
Source: JMIR Res Protoc. 2021 Jul 28;10(7):e25177. doi: 10.2196/25177 (PMC8367153; doi:10.2196/25177)
Supplement: Multimedia Appendix 3 [file resprot_v10i7e25177_app3.pdf]

Appendix 3. Functions of DHI-Healthcare Providers RECODED for COMPREHENSION

ORIGINAL		RE-CODED	V1	V2	V3	V4	V5
**2.1 Client Identification & Registration**	Verify client unique identity	**2.1 Patient identification & registration**	Verify a patient's details	Confirm a patient's details (e.g demographics)	Verify a patient's details on a new form	Verify a patient's personal details (e.g. new patient registration)	
	Enrol client for a health service or care planning activity		Register a patient for a service or care planning activity	Enrol a patient into a service or care planning activity	Make a clinical appointment		
**2.2 Client Health Records**	Longitudinal tracking of client's health status & services	**2.2 Patient Health Records**	Track a patient's health status &/or service use over time	Track a patient's condition &/or clinical service use over time	Track a patient's condition and/or clinical service use over time		
	Manage the client's UNSTRUCTURED clinical records		Manage a patient's unstructured clinical records (ie this means electronic notes that aren't organised in any organised/meaningful way)	Manage a patient's free-text, clinical progress notes	Enter a patient's free-text clinical progress notes		
	Manage the client's STRUCTURED clinical record		Manage a patient's structured clinical records (ie this means electronic notes that are organised and categorised according to standardised codes, checkboxes, and dropdowns)	Manage a patient's clinical progress using standardised coding, checkboxes, and dropdown menus	Record or code a patient's condition using standardised coding, checkboxes, and dropdown menus		
	Routine health indicator data collection & management		Collect & manage routine indicators of patient's health status	Collect & manage clinical indicators of the status of a patient's condition	Record and flag indicators or changes in a patient's condition	Record and/or flag indicators of change in a patient's condition	
**2.3 Healthcare Provider Decision Support**	Provide prompts and alerts according to a specific protocol	**2.3 Clinician decision-support**	Provide prompts and alerts according to a specific protocol	Provide prompts and alerts that follow specific protocols (e.g. decision support flow diagrams)	Prompt and alert me using software that supports clinicial decision making	Prompt my thinking using software that supports clinical decision-making	
	Provide a checklist according to a specific protocol		Provide a checklist according to a specific protocol	Provide a checklist that follow's a specific protocol	Provide a digital checklist of clinical procedures	Provide me a digital checklist of clinical procedures to follow	
	Screen clients by risk or, other health status		Screen patients by risk or, other health status indicators	Screen patients	Screen my patients		
**2.4 Telemedicine**	Consultations between remote client and healthcare provider	**2.4 Telemedicine**	Consultations between a remotely located patient and clinician	Conduct consultations with remotely located patients	Conduct remote consultations		
	Remote monitoring of client health, or diagnostic data by healthcare provider		Remote monitoring of patient's health status, or diagnostic data by clinician	Remotely monitor a patient's condition, or track diagnostic data	Remotely monitor or track a patient's condition		
	Transmission of health-related data to healthcare provider		Transmission of health-related data to clinician	Transmit health-related data about my patient back to me	Send me data about my patient's condition		
	Consultations for case-management between healthcare providers		Consultations for case-management between clinicians	Conduct consultations/meetings with other clinicians for case-management	Conduct case consultations with other clinicians		
**2.5 Healthcare Provider Communication**	Communication from healthcare providers to supervisor	**2.5 Clinician communications**	Communication from clinicians to their manager or supervisor	To communicate with a manager or supervisor	Communicate with a manager or supervisor		
	Communication and performance feedback to healthcare providers		Communication and performance feedback to clinicians	To communicate or provide feedback to me about my clinical performance	Provide me with feedback about my clinical performance		
	Transmit routine news and workflow notifications to healthcare providers		Transmit routine updates and workflow notifications to clinicians	Transmit routine updates and workflow notifications to me	Send me routine updates and workflow notifications		
	Transmit non-routine health event alerts to healthcare providers		Transmit non-routine health event alerts about a patient to clinician	Transmit non-routine alerts about a patient's condition status to me	Send non-routine or unexpected health event alerts about a patient		
	Peer-group for healthcare providers		Online peer-to-peer group for clinicians	Online peer-to-peer communication group for clinicians	Online peer communication groups	Create online peer communication groups for clinicians	Utilise online peer communication groups for clinicians
**2.6 Referral Coordination**	Coordinate emergency response and transport	**2.6 Referral Coordination**	Coordinate emergency responses and patient transport	Coordinate emergency responses and transport for a patient	Coordinate emergency responses and transport for a patient	Coordinate emergency responses and/or transport for a patient	
	Manage referrals between points of service with health sector		Manage referrals between different points of service in the health system	Manage referrals between different points of services in the health system	Manage health services referrals e.g. to other clinicians	Manage health services referrals or reports (e.g. to other clinicians)	
	Manage referrals between health and other sectors		Manage referrals between the health system and other sectors	Manage referrals between the health system and other sectors	Manage referrals to external bodies e.g. government services	Manage referrals or reports to external bodies e.g. government services	
**2.7 Health Worker Activity Planning & Scheduling**	Identify clients in need of services	**2.7 Clinician workflow coordination**	Identify patients in need of services	Identify patients in need of a health service	Identify patients in need of a health service		
	Schedule healthcare provider's activities		Schedule clinician's activities	Schedule my clinical activities	Schedule my clinical activities		
**2.8 Healthcare Provider Training**	Provide training content to healthcare providers	**2.8 Clinician training**	Provide training or educational content to clinicians	Provide training or educational content to me	Provide training or educational content to me	Provide me with training or educational content	
	Assess capacity of healthcare providers		Assess capacity, or performance of clinicians	To assess my clinical capacity, or performance	Assess my clinical capacity	Assess my clinical capacity, or performance	
**2.9 Prescription & Medication Management**	Transmit or track prescription orders	**2.9 Prescription & medication management**	Transmit or track prescription orders	Transmit or track prescriptions	Track prescription orders		
	Track clients' medication consumption		Track patient's medication consumption				
**2.10 Laboratory & Diagnostic Imaging Management**	Report adverse drug events	**2.10 Pathology & Imaging Management**	Report drug adverse events	Report adverse medication events			
	Transmit diagnostic result to healthcare providers		Transmit diagnostic results to clinicians	Transmit diagnostic results	Send diagnostic imaging results to me e.g. scans	Send me diagnostic imaging results (e.g. scans)	
	Transmit and track diagnostic orders		Transmit and track diagnostic orders	Transmit and track diagnostic orders (i.e. scans)	Track diagnostic imaging orders		
	Capture diagnostic results from digital devices		Capture diagnostic results from digital devices				
	Track biological specimens		Track pathology specimens	Track pathology e.g. blood tests			
