# Supplementary material for: Digital Health Interventions in Physiotherapy: Development of Client and Health Care Provider Survey Instruments
Source: JMIR Res Protoc. 2021 Jul 28;10(7):e25177. doi: 10.2196/25177 (PMC8367153; doi:10.2196/25177)
Supplement: Multimedia Appendix 4 [file resprot_v10i7e25177_app4.pdf]

Digital Health Intervention Willingness – Client Survey – DHIW-C

Using Digital Health Technology in [insert context]

Thank you for your interest in our study.

This study aims to explore the potential for **digital health technologies** (e.g. smartphones, apps, electronic health records, wearable sensors, digital video...to name a few) to support how [xxxxxxx conditions are managed between you and a xxxxxxxx].

Willingness to Use Digital Health Technologies

Digital technologies (e.g. smartphones, apps, websites, electronic health records, wearables, etc) can be used for a range of different **PURPOSES** as part of your [insert discipline area] care. These are listed below*.

We are interested in understanding HOW WILLING YOU ARE to use digital health technology to support you and your [insert healthcare provider discipline] to manage **your [insert condition].**

**The following items are adaptations of items in the World Health Organisation's Classifications of Digital Health Interventions v1.0*

©Merolli et al 2020

For each **purpose** listed below, when considering managing your **[insert condition]:**

HOW WILLING **ARE YOU** to use digital technology to...

	Not at all willing	A little bit	Somewhat	Quite a bit	Very much willing
Send me urgent health alerts that people living with my condition need to know (e.g. medication product recalls, etc)	○	○	○	○	○
Send me health information of interest for people living with my condition (e.g. about new treatments, research, etc)	○	○	○	○	○
Send personalised alerts and reminders relevant specifically to me (e.g about services I've booked or have coming up)	○	○	○	○	○
Send me health test results, or tell me results are available	○	○	○	○	○
Send me general news or information about good health or healthy living	○	○	○	○	○
Send me general health alerts (e.g. about environmental factors impacting my ability to exercise- weather, air quality, etc)	○	○	○	○	○
Communicate online with other peer groups of people living with my condition	○	○	○	○	○
Access my own medical records	○	○	○	○	○
Self monitor my condition or diagnosis-related information	○	○	○	○	○
Actively collect information about my condition or injury status and record it	○	○	○	○	○
Allow me to collect and provide feedback about the health system	○	○	○	○	○

©Merolli et al 2020

	Not at all willing	A little bit	Somewhat	Quite a bit	Very much willing
Allow me to report urgent public health events/issues that people living with my condition need to know	○	○	○	○	○
Look up health information	○	○	○	○	○
Send or manage any 'out of pocket' payments I may need to pay	○	○	○	○	○
Send or manage vouchers/coupons I might have for health services (e.g travel vouchers, etc)	○	○	○	○	○
Send or manage rewards or incentives I have to use health services	○	○	○	○	○

Can you think of any other **purposes** not identified in the previous table that you think digital health technologies could help you with managing a xxxxxx condition?
(Please specify below)

____

____

____

____

____

©Merolli et al 2020
