# Supplementary material for: Digital Health Interventions in Physiotherapy: Development of Client and Health Care Provider Survey Instruments
Source: JMIR Res Protoc. 2021 Jul 28;10(7):e25177. doi: 10.2196/25177 (PMC8367153; doi:10.2196/25177)
Supplement: Multimedia Appendix 5 [file resprot_v10i7e25177_app5.pdf]

Digital Health Intervention Willingness – Healthcare Provider Survey – DHIW-HCP

Using Digital Health Technology in [insert healthcare discipline] Practice

Thank you for your interest in our study.

This study aims to explore the potential for **digital health technologies** (e.g. smartphones, apps, electronic health records, wearable sensors, digital video...to name a few) to support the delivery of [insert healthcare discipline] care to patients with [insert condition(s)].

We are interested in your opinions of how these technologies can support the assessment and management of patients.

Willingness to Use Digital Health Technologies

Digital technologies (e.g. smartphones, apps, electronic health records, wearable sensors, digital video, etc) can be used to support a range of different **FUNCTIONS** in health care. These are listed below*.

We are interested in understanding **HOW WILLING YOU ARE** to use digital health technology to support you in your clinical role as a [insert healthcare discipline].

**The following items are adaptations of items in the World Health Organisation's Classifications of Digital Health Interventions v1.0*

For each FUNCTION listed below, **HOW WILLING ARE YOU** to use digital technology to...

	Not at all willing	A little bit	Somewhat	Quite a bit	Very much willing
Verify a patient's personal details (e.g. new patient registration)	○	○	○	○	○
Make a clinical appointment	○	○	○	○	○
Track a patient's condition and/or clinical service use over time	○	○	○	○	○
Enter a patient's free-text clinical progress notes	○	○	○	○	○
Record or code a patient's condition using standardised coding, checkboxes, and dropdown menus	○	○	○	○	○
Record and/or flag indicators of change in a patient's condition	○	○	○	○	○
Prompt my thinking using software that supports clinical decision-making	○	○	○	○	○
Provide me a digital checklist of clinical procedures to follow	○	○	○	○	○
Screen my patients	○	○	○	○	○
Conduct remote consultations	○	○	○	○	○
Remotely monitor or track a patient's condition	○	○	○	○	○
Send me data about my patient's condition	○	○	○	○	○
Conduct case consultations with other clinicians	○	○	○	○	○
Communicate with a manager or supervisor	○	○	○	○	○
Provide me with feedback about my clinical performance	○	○	○	○	○
	Not at all willing	A little bit	Somewhat	Quite a bit	Very much willing

©Merolli et al 2020

Send me routine updates and workflow notifications	○	○	○	○	○
Send me non-routine or unexpected health event alerts about a patient	○	○	○	○	○
Utilise online peer communication groups for clinicians	○	○	○	○	○
Coordinate emergency responses and/or transport for a patient	○	○	○	○	○
Manage health service referrals or reports (e.g. to other clinicians)	○	○	○	○	○
Manage referrals or reports to external bodies (e.g. government services)	○	○	○	○	○
Identify patients in need of a health service	○	○	○	○	○
Schedule my clinical activities	○	○	○	○	○
Provide me with training or educational content	○	○	○	○	○
Assess my clinical capacity, or performance	○	○	○	○	○
Track prescription orders	○	○	○	○	○
Track patients' medication consumption	○	○	○	○	○
Report adverse medication events	○	○	○	○	○
Send me diagnostic imaging results (e.g. scans)	○	○	○	○	○
Track diagnostic imaging orders	○	○	○	○	○
Capture diagnostic results from digital devices	○	○	○	○	○
Track pathology (e.g. blood tests)	○	○	○	○	○

©Merolli et al 2020

Can you think of any other **functions** not identified in the previous table that you think digital health technologies could assist with?
(Please specify below)
